# Supplementary material for: CD45 pre-exclusion from the tips of T cell microvilli prior to antigen recognition
Source: Nat Commun. 2021 Jun 23;12:3872. doi: 10.1038/s41467-021-23792-8 (PMC8222282; doi:10.1038/s41467-021-23792-8)
Supplement: Supplementary file 1 — Supplementary Information [file 41467_2021_23792_MOESM1_ESM.pdf]

Supplementary Information

Jung *et al.*

Title: CD45 pre-exclusion from the tips of T cell microvilli prior to antigen recognition

Supplementary Figures

Supplementary Fig. 1

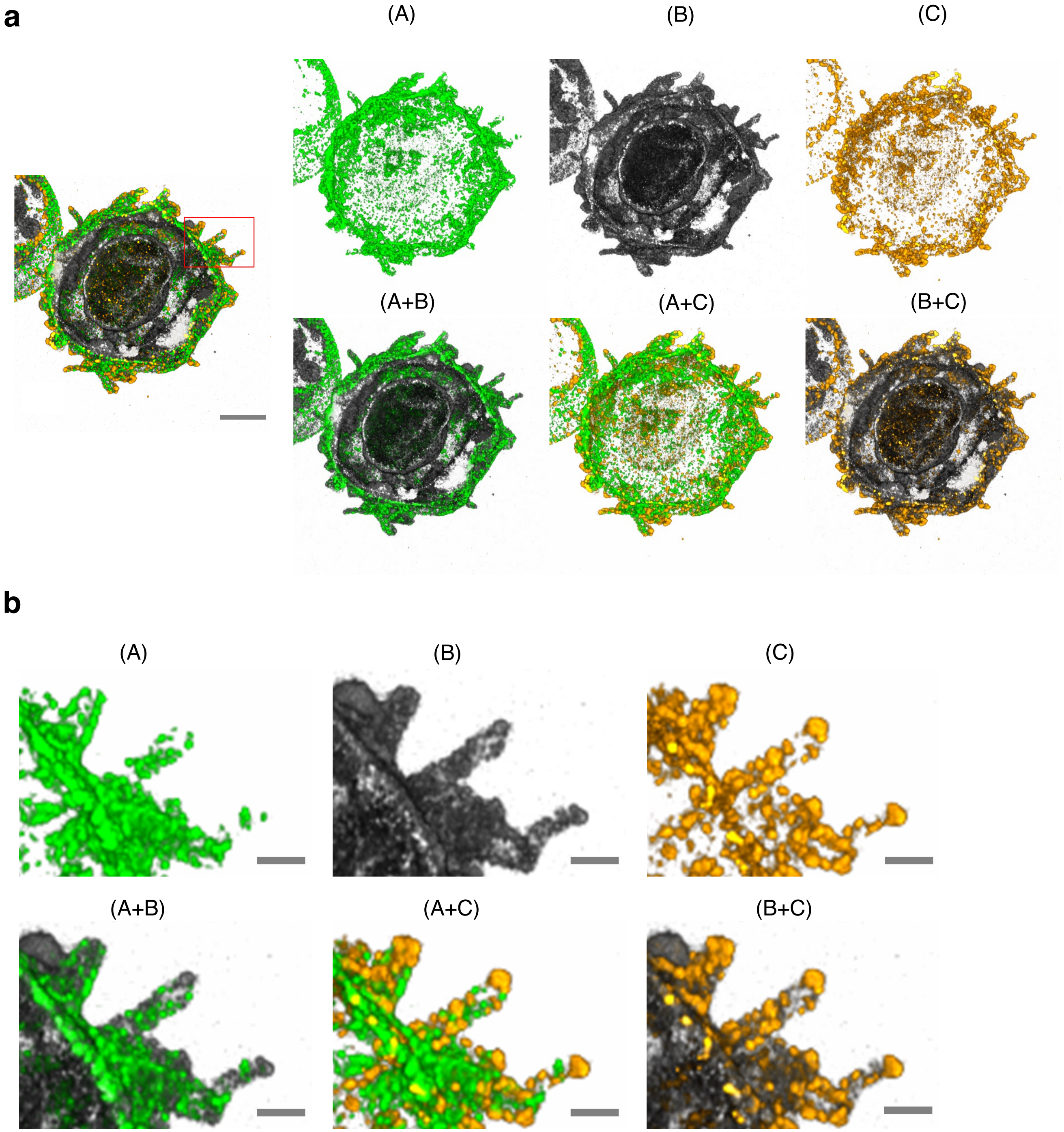

(A) CD45; (B) Membrane; (C) L-sel

Supplementary Fig 1. Pre-exclusion of CD45 at the tips of MV on human resting CD4$^+$ T cells. **a** Representative reconstructed 3D 4x-ExM-Airyscan images of a human CD4$^+$ T cell labeled with anti-CD45-AF488 (green), FM 4-64FX membrane dye (gray) and anti-L-sel-AF568 (dark yellow). Individual channel images of CD45 (A), membrane (B), and L-sel (C) (right, upper panels), merged images of (A) and (B), (A) and (C), (B) and (C) (right, lower panels), and merged image of (A), (B) and (C) (left) are displayed. These images are representative of 9 cells analyzed. Scale bar in a: 2 μm. **b** Magnified images of CD45 (A), membrane (B), and L-sel (C) (upper panels), merged images of (A) and (B), (A) and (C), and (B) and (C) (lower panels) of the area marked by the red rectangle in a. Scale bars: 500 nm.

Supplementary Fig. 2

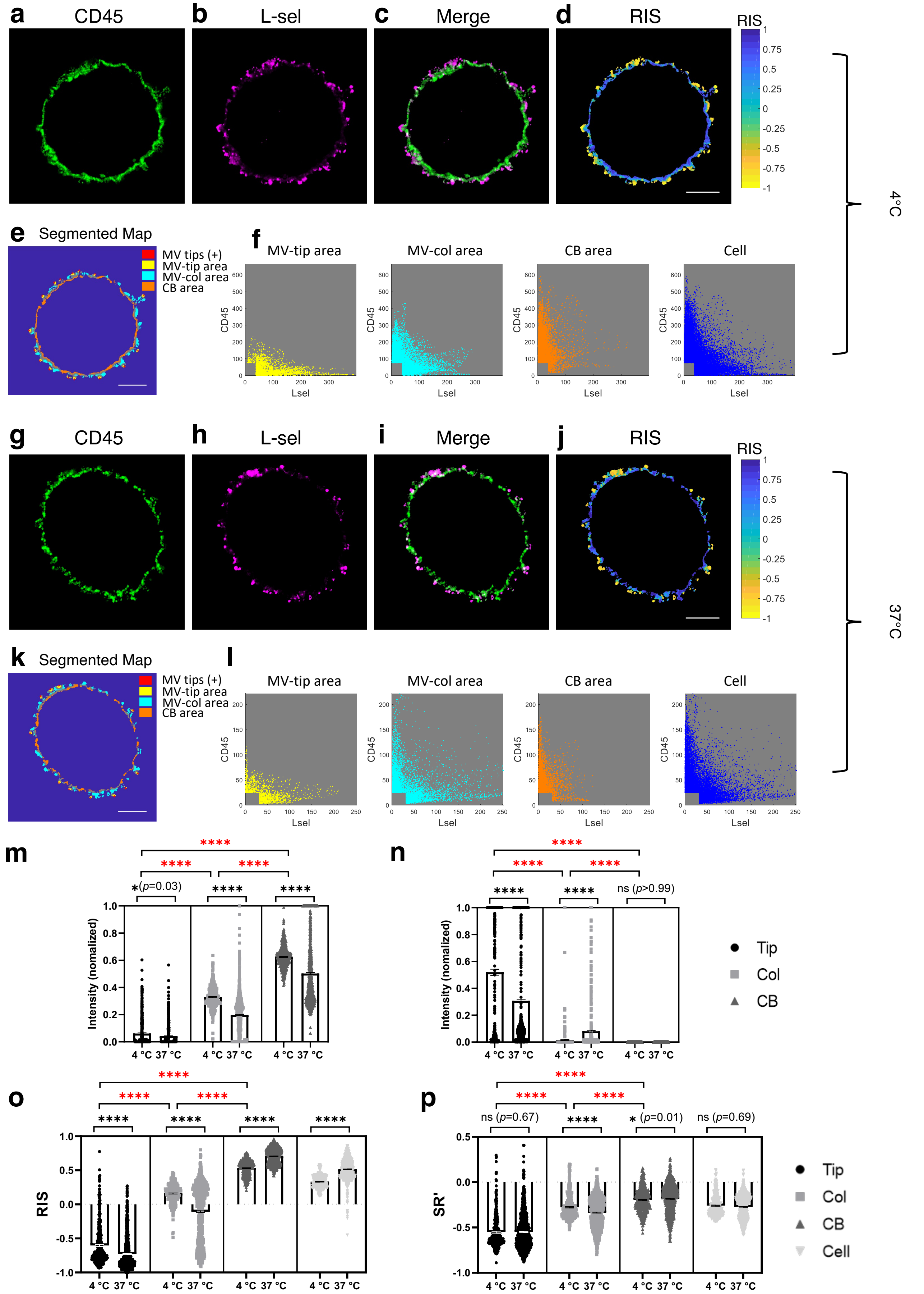

Supplementary Fig. 2. CD45 and L-sel in human resting CD4 T^+ cells. **a-b** Representative 4x-ExM-Airyscan images of a $CD4^+$ T cell labeled with anti-CD45-AF488 (green; a) and anti-L-sel-AF568 (magenta; b). **c** The merged image of a and b. Images in a-c are representative of 20 cells in three independent experiments. **d** The RIS image between a and b. **e** The segmented map of the cell in a-c for positions of the individual MV tips (MV tips, red cross), MV-tip area (yellow), MV-column area (MV-col area, cyan), and cell body area (CB area, orange). **f** The intensity scatter plots for L-sel and CD45 within the MV-tip (yellow), MV-col (cyan), CB (orange), and entire cell (Cell, blue) areas of the cell shown in e. **g-h** Representative 4x-ExM-Airyscan images of a $CD4^+$ T cell labeled with anti-CD45-AF488 (green; g) and anti-L-sel-AF568 (magenta; h). **i** The merged image of g and h. Images in g-i are representative of 54 cells in three independent experiments. **j** The RIS image between g and h. **k** The segmented map of the cell in g-i described as e. Scale bars in d, e, j and k: 2 μm. **l** The intensity scatter plots for L-sel and CD45 within the segmented area shown in k described as f. **m-n** The median CD45 (m) and L-sel (n) intensities (normalized) within the segmented areas, MV-tip (Tip, black circle), MV-col (Col, gray square), CB (CB, dark gray triangle) areas of each z-plane images (20 z-plane images per cell) of CD4 T cells (labeled at 4 °C: 20 cells; labeled at 37 °C: 54 cells). **o-p** The median RIS values (o) and the mean SR′ values (p) within the segmented areas, MV-tip area, MV-col area, CB area and entire cell (Cell, light gray downward-triangle) of each cells described as m-n. Each dot represents data collected from a z-plane image. Bars represent the mean and error bars represent the SE. *p*-values (^{ns} (not significant), $p > 0.05$; *, $p \leq 0.05$; ****, $p \leq 0.000$) were calculated by two tailed Wilcoxon matched-pairs signed rank test (red) or by two tailed Mann Whitney test (black). Source data for m-p are provided as a Source Data file.

Supplementary Fig. 3

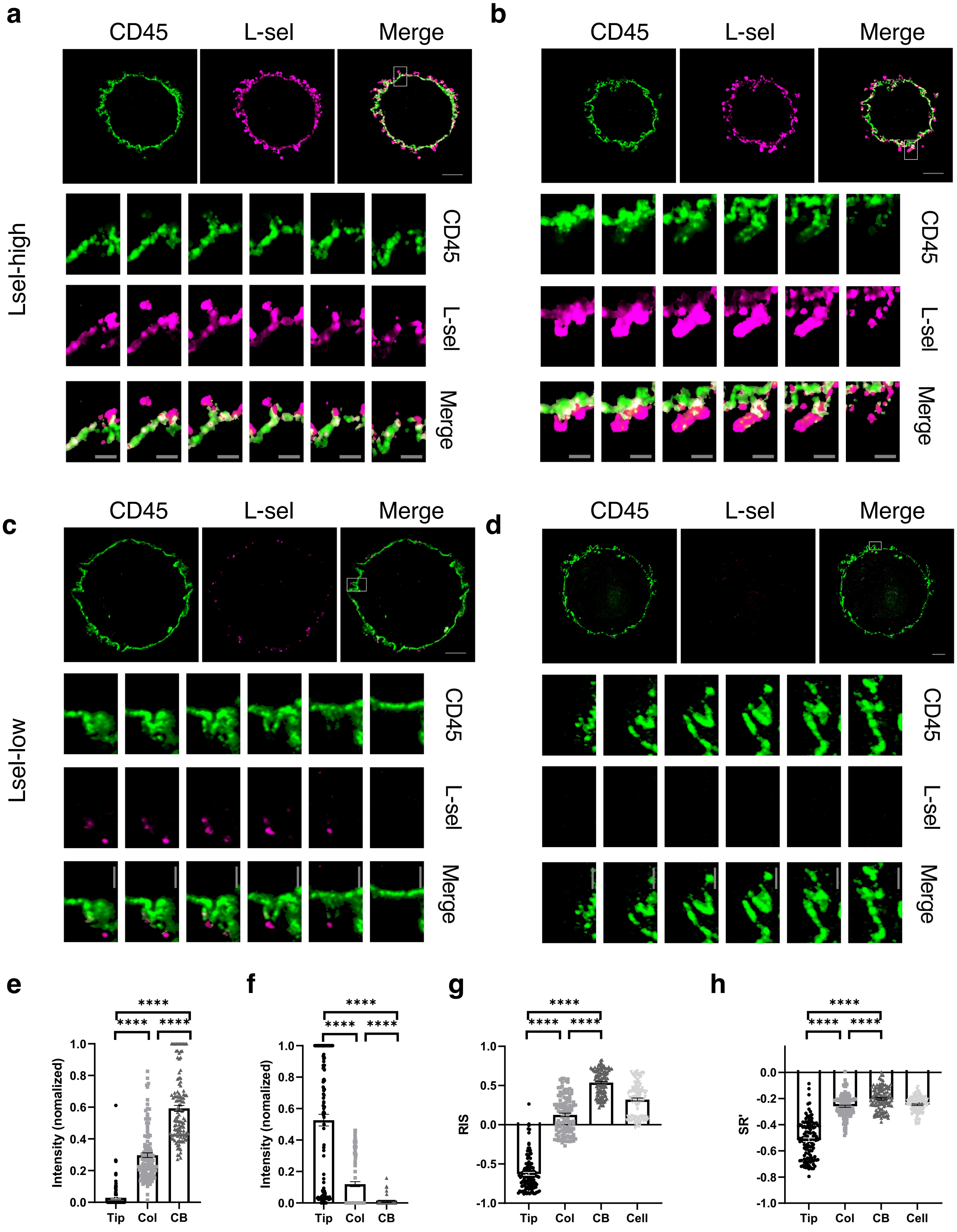

Supplementary Fig. 3. CD45 pre-exclusion from MV tips of human L-sel-high and -low effector $CD4^+$ T cells. **a-b** Representative 4x-ExM-Airyscan images of CD45 (green), L-sel (magenta), and the merged images of these two expressed on $L\text{-sel}^{high}$ *in vitro*-differentiated effector $CD4^+$ T cells (top panels). The magnified z-stack series of images with a step size of 125 nm marked by a square in the merged images are displayed in the bottom panels. **c-d** Analysis similar to a and b of $L\text{-sel}^{low}$ effector $CD4^+$ T cells. Scale bars: 2 μm in the merged images; 500 nm in the magnified merged images. Images in a-d are representative of three independent experiments. **e-f** The median CD45 (e) and L-sel (f) intensities (normalized) within the segmented areas (MV-tip area (Tip, black circle), MV-col area (Col, gray square), CB area (CB, dark gray triangle)) of each z-plane images (20 z-plane images per cell) of the $L\text{-sel}^{high}$ effector $CD4^+$ T cells (6 cells). Each dot represents data collected from a z-plane image. Bars represent the mean and error bars represent the SE. **g** The median RIS values within the segmented areas or entire cell (Cell, light gray downward-triangle) described as e. **h** The mean SR′ values within the segmented areas described as g. *p*-values (****, $p \leq 0.0001$) were calculated by two tailed Wilcoxon matched-pairs signed rank test. Source data for e-h are provided as a Source Data file.

Supplementary Fig. 4

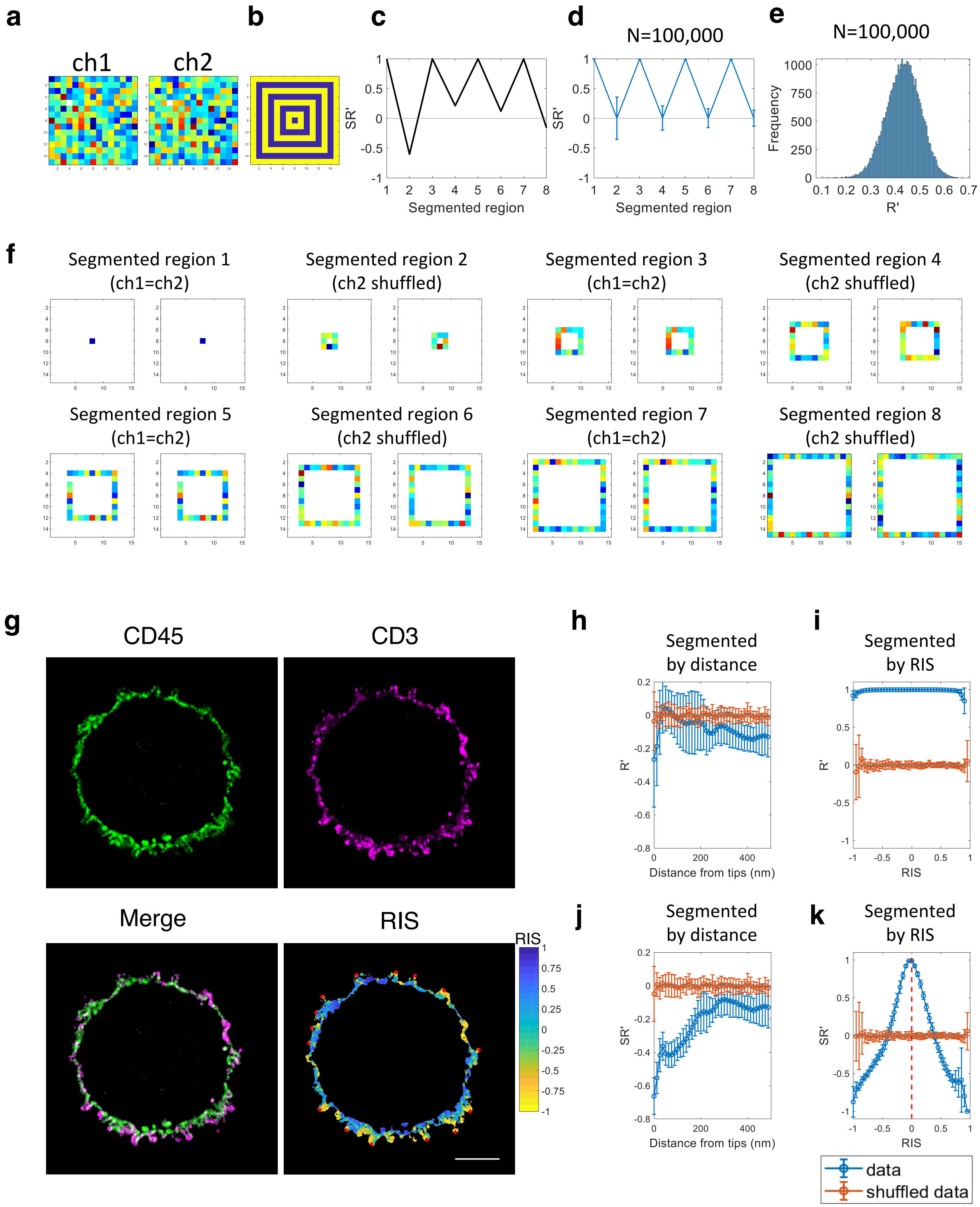

Supplementary Fig. 4. The segment correlation coefficient (SR′). **a-b** An example of simulated images for channel 1 (ch1) and channel 2 (ch2). Ch1 image was generated by assigning random intensities (mean=100; SD=2). Ch2 pixel intensities were identical with ch1 in the blue-marked area in b, and the rest of intensities (the yellow-marked area in b) were randomly shuffled and reassigned. **c** SR′ for the eight individual segmented data sets presented in f. **d** The mean SR′s for the eight individual segmented data sets similarly presented in f from 100,000 simulated data points as a. **e** Distribution of the Pearson's correlation coefficient, R′, between ch1 and ch2 of 100,000 simulated data used in d. **f** The individual segmented area of a. **g** A representative example of 4x-ExM-Airyscan images of CD45 (green) and CD3 (magenta) of a human resting CD4 T cell and the merged and RIS images between the two images. The red dots in the RIS image represent the locations of MV tips. Scale bar: 2 μm. **h** The mean R′ calculated from 20 frames of the cell presented in g segmented by distance from MV tips was plotted as a function of the distance (blue). The mean R′ calculated after the two-channel intensities were randomly shuffled (orange). **i** The mean R′ calculated for the data set in h segmented by RIS values with a step-size of 0.05 was plotted as a function of the RIS. The mean R′ calculated after the two-channel intensities were randomly shuffled (orange). **j** The mean SR′ calculated from 20 frames of the cell presented in g segmented by distance from MV tips was plotted as a function of the distance (blue). The mean SR′ calculated after the two-channel intensities were randomly shuffled (orange). **k** The mean SR′ calculated for the data set in **j** segmented by RIS values with a step-size of 0.05 was plotted as a function of the RIS. The mean SR′ calculated after the two-channel intensities were randomly shuffled (orange). The mean RIS value (zero) of the 20 frames of RIS images is marked with a red-vertical line. Error bars represent SD. Source data for c-e and h-k are provided as a Source Data file.

Supplementary Fig. 5

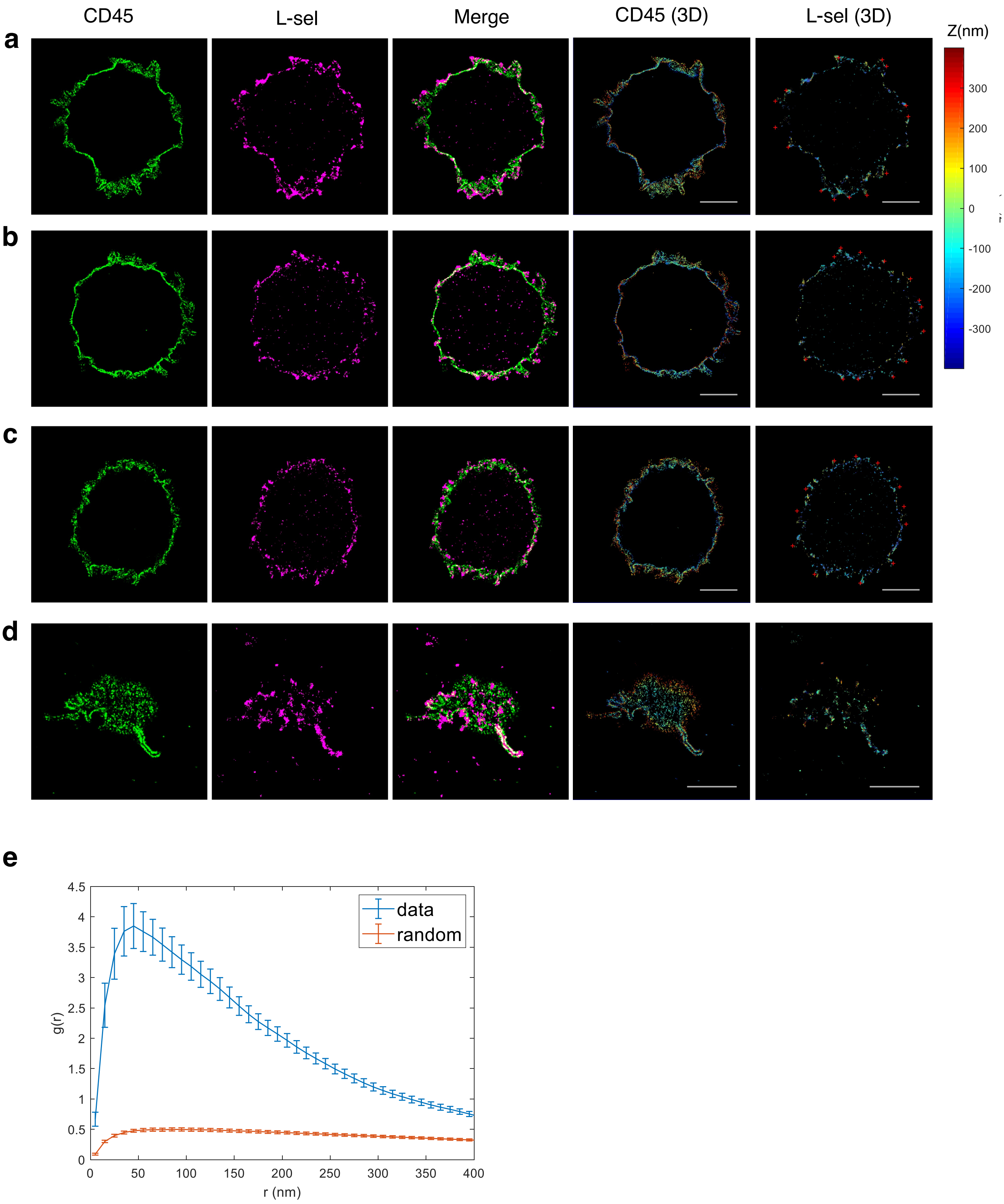

Supplementary Fig. 5. STORM images of CD45 and L-sel expressed on human resting $CD4^+$ T cells. **a-d** Representative STORM images of AF647-CD45 (green) and AF568-L-sel (magenta) and the merged images. 4 representative human $CD4^+$ T cells selected among 26 cells (3 left rows) from three independent experiments. Images were rendered by pixel size of 10nm. For each channel, single molecules collected from 30,000 frames were reconstructed. The localizations of the z-dimension of the CD45 and L-sel molecules were color-coded (2 right rows). Images were acquired at 1.5~2 μm above the glass surface (a-c) or at the glass surface (d). Scale bars: 2 μm. **e** The mean pair-correlation, g(r), (n=25 cells) as a function of radius (r) (blue) was plotted. The mean pair-correlation calculated after localizations were randomly assigned was plotted (orange). The tip positions used for the pair-correlation analysis are marked with red-cross on the L-sel (3D) images (right) in a-c. The cell shown in d was excluded for the pair-correlation analysis. Error bars represent SE. Source data for e are provided as a Source Data file.

Supplementary Fig. 6

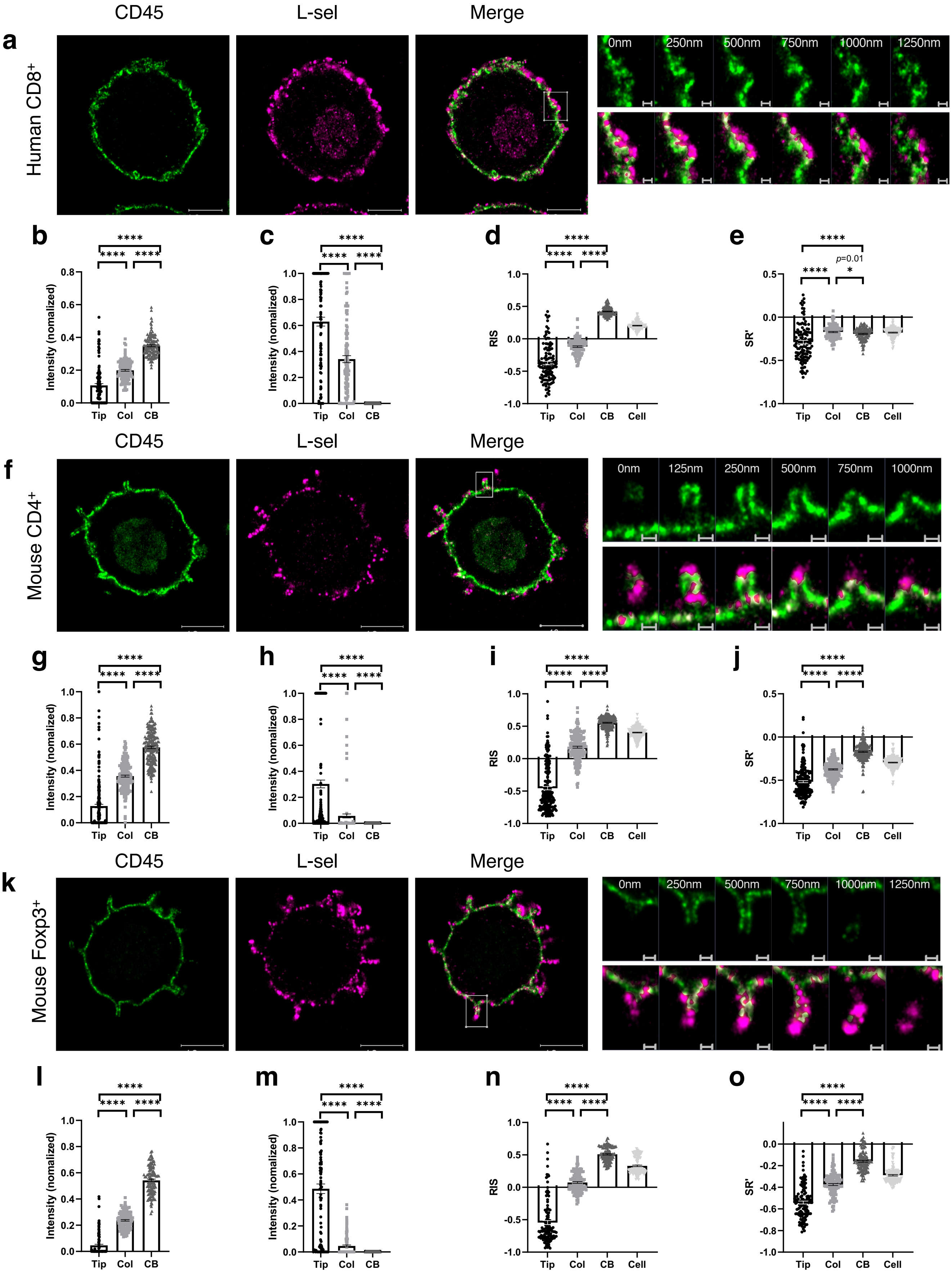

Supplementary Fig. 6. CD45 pre-exclusion from MV tips in various types of human or mouse T cells. **a** Representative 4x-ExM-Airyscan images of CD45 (green) and L-sel (magenta) and the merged image expressed on a human $CD8^+$ T cell. Six z-stack images (step size 250 nm) of the area marked by a square in the merged image were magnified (right). Scale bars: 2.5 μm in left 3 images; 250 nm in the magnified images. **b-e** The medians of the CD45 intensities (b), the L-sel intensities (c) and the RIS values (d), or the mean SR′ values (e) within the segmented areas (MV-tip area (Tip, black circle), MV-col area (Col, gray square), CB area (CB, dark gray upward-triangle)) or entire cell (Cell, light gray downward-triangle) of each z-plane images (20 z-plane images per cell) of human $CD8^+$ T cells (6 cells). Each dot represents data collected from a z-plane image. Bars represent the mean and error bars represent the SE. **f** Similar analysis of CD45 (green) and L-sel (magenta) expressed on a mouse $CD4^+$ T cell as a. **g-j** The medians of the CD45 intensities (g), the L-sel intensities (h), and the RIS values (i), or the mean SR′ values (j) of mouse $CD4^+$ T cells (10 cells) were similarly analyzed as b-e, respectively. **k** Similar analysis of CD45 (green) and L-sel (magenta) expressed on a mouse $CD4^+$*Foxp3*-YFP^+ Treg cell. **l-o** The medians of the CD45 intensities (l), the L-sel intensities (m), and the RIS values (n), or the mean SR′ values (o) of *Foxp3*$^+$ T cells (6 cells) were similarly analyzed as b-e, respectively. Images are representative of two independent experiments. *p*-values (*, $p \leq 0.05$; ****, $p \leq 0.0001$) were calculated by two tailed Wilcoxon matched-pairs signed rank test. Source data for b-e, g-j, and l-o are provided as a Source Data file.

Supplementary Fig. 7

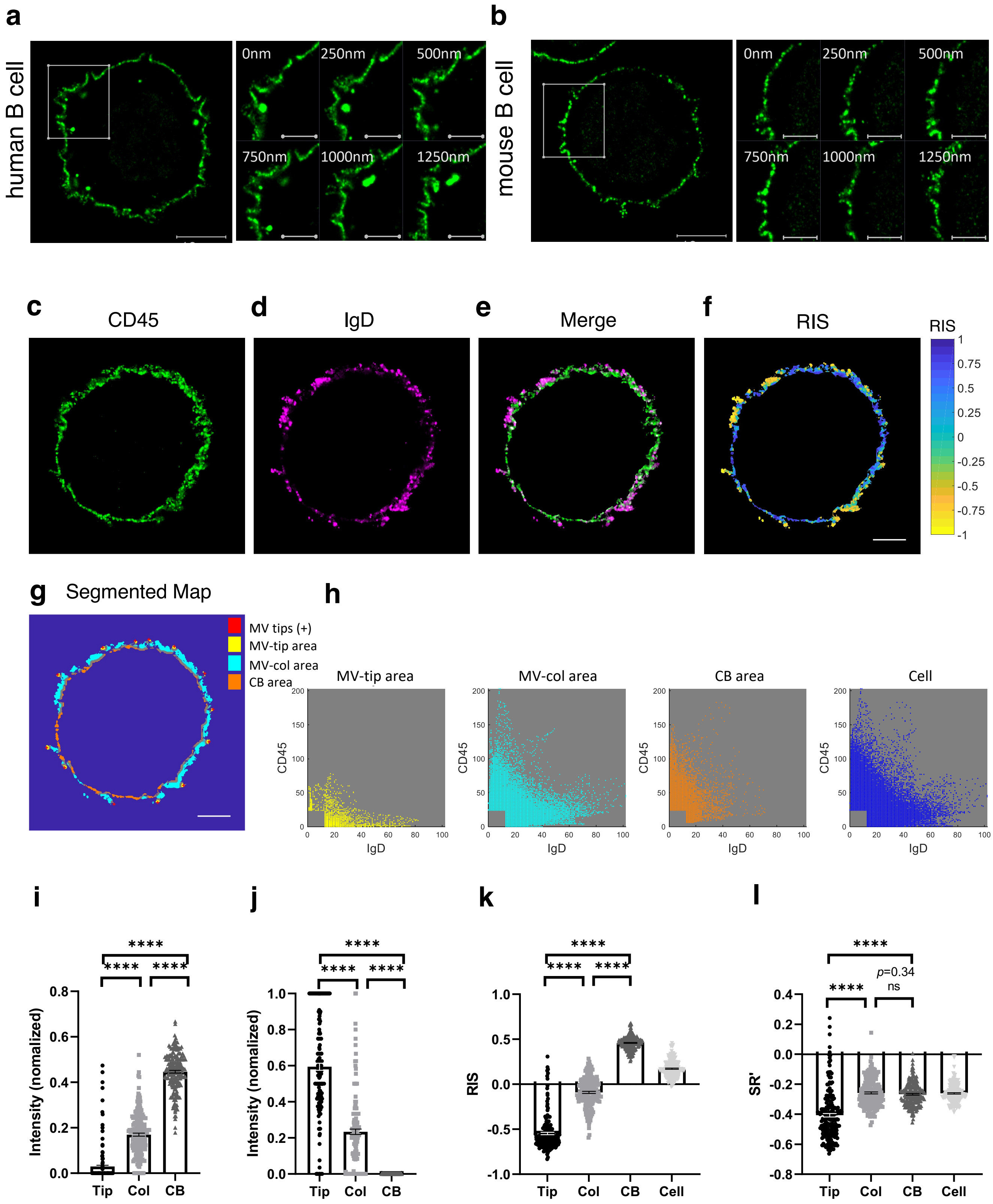

Supplementary Fig. 7. CD45 pre-exclusion and highly enriched membrane-bound IgD from MV-tips in B cells. **a-b** Representative 4x-ExM-Airyscan images of CD45 (green) expressed on human (a) and mouse (b) B cells. Six z-stack images (step size 250 nm) of the area marked by a square in the merged image were magnified (right). Scale bars: 2.5 μm in the left images; 1.25 μm in the magnified images. **c-e** Representative 4x-ExM-Airyscan images of a human B cell labeled with anti-CD45-AF488 (green; c), anti-IgD-CF633 (magenta; d), and the merged image (e). Images in a-e are representative of three independent experiments. **f** The RIS image between c and d. **g** The segmented map of the cell in c-e for the positions of the individual MV tips (MV tips, red cross), MV-tip area (yellow), MV-column area (MV-col area, cyan), and cell body area (CB area, orange). Scale bars in f and g: 2 μm. **h** The intensity scatter plots for IgD and CD45 within the MV-tip area (yellow), MV-col area (cyan), CB area (orange), and entire cell area (Cell, blue) of the cell shown in g. **i-l** The medians of the CD45 intensities (i), the IgD intensities (j), and the RIS values (k), or the mean SR′ values (l) within the segmented areas (MV-tip area (Tip, black circle), MV-col area (Col, gray square), CB area (CB, dark gray upward-triangle)) or entire cell (Cell, light gray downward-triangle) of each z-plane images (20 z-plane images per cell) of B cells (10 cells). Each dot represents data collected from a z-plane image. Bars represent the mean and error bars represent the SE. *p*-values (^{ns}, $p > 0.05$; ****, $p \leq 0.0001$) were calculated by two tailed Wilcoxon matched-pairs signed rank test. Source data for i-l are provided as a Source Data file.

Supplementary Fig. 8

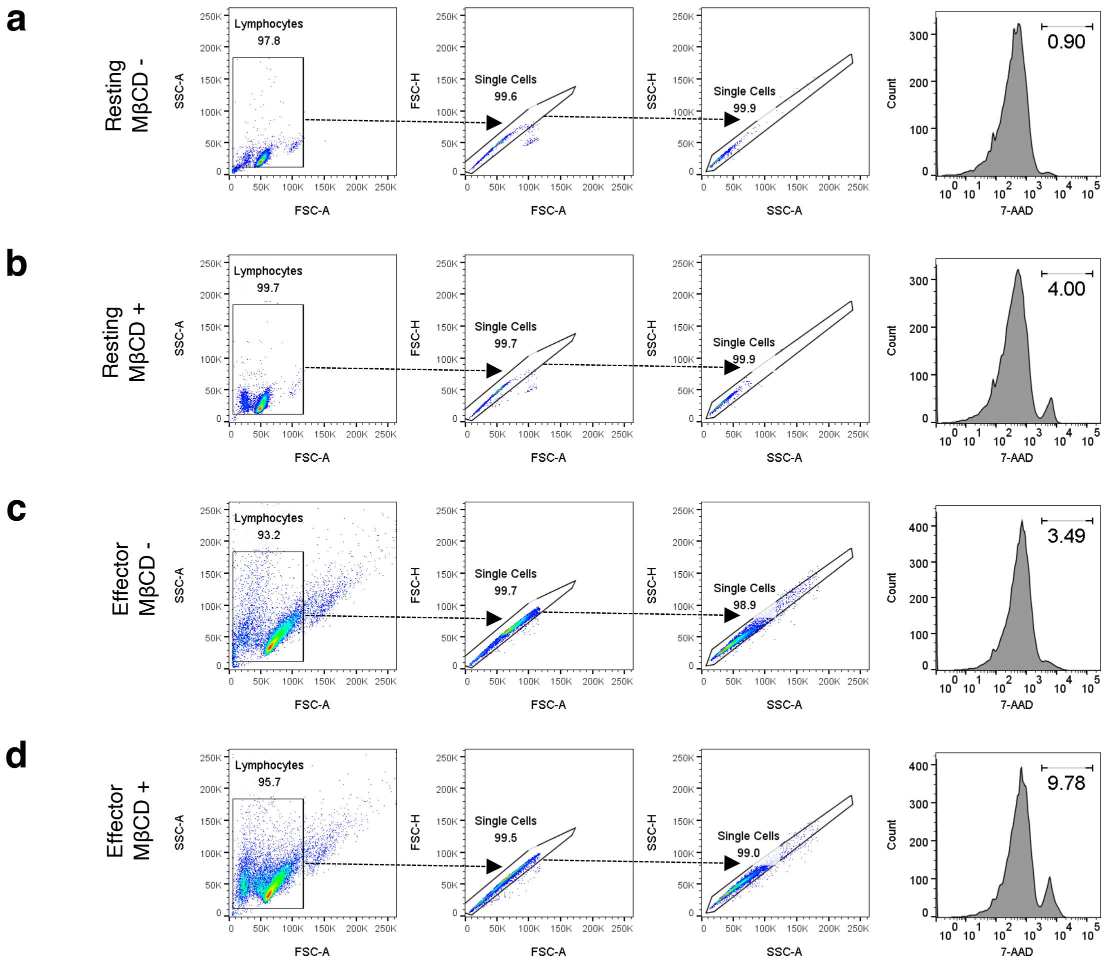

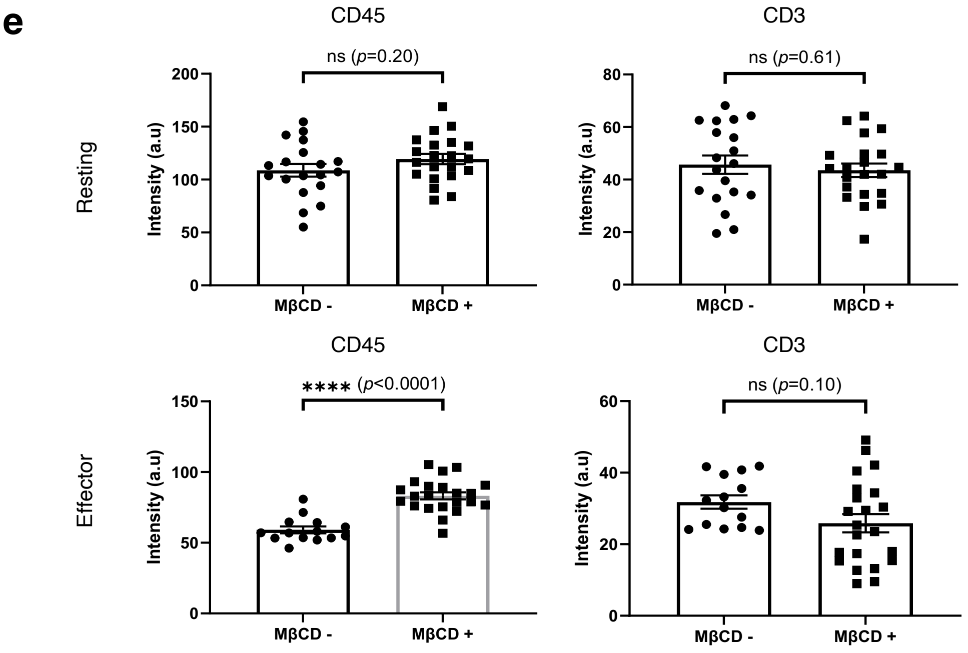

Supplementary Fig. 8. Effect of MβCD on human $CD4^+$ T cells. **a-d** The effect of MβCD treatment on the viability of human resting (a-b) and effector (c-d) $CD4^+$ cells was analyzed by flow cytometry. Single cells were gated using forward vs. side scatter (FSC vs. SSC) parameters (left 3 panels) and $7AAD^+$ positive dead cells were counted (right panels) by FlowJo software. The percentages of gated cells were displayed in each graph. **e** The mean CD45 and CD3 intensities of human resting (upper panels) and effector (lower panels) $CD4^+$ T cells untreated (MβCD –, 0 mM, black circle) or treated with MβCD (MβCD +, 10 mM, black square). Each dot represents data collected from a cell (MβCD – resting: 19 cells; MβCD + resting :21 cells; MβCD – Effector: 14 cells; MβCD + Effector: 22 cells from three independent experiments). Bars represent the mean and error bars represent the SE. *p*-values (^{ns}, $p > 0.05$; ****, $p \leq 0.0001$) were calculated by two tailed Mann Whitney test. Source data for e are provided as a Source Data file.

Supplementary Fig. 9

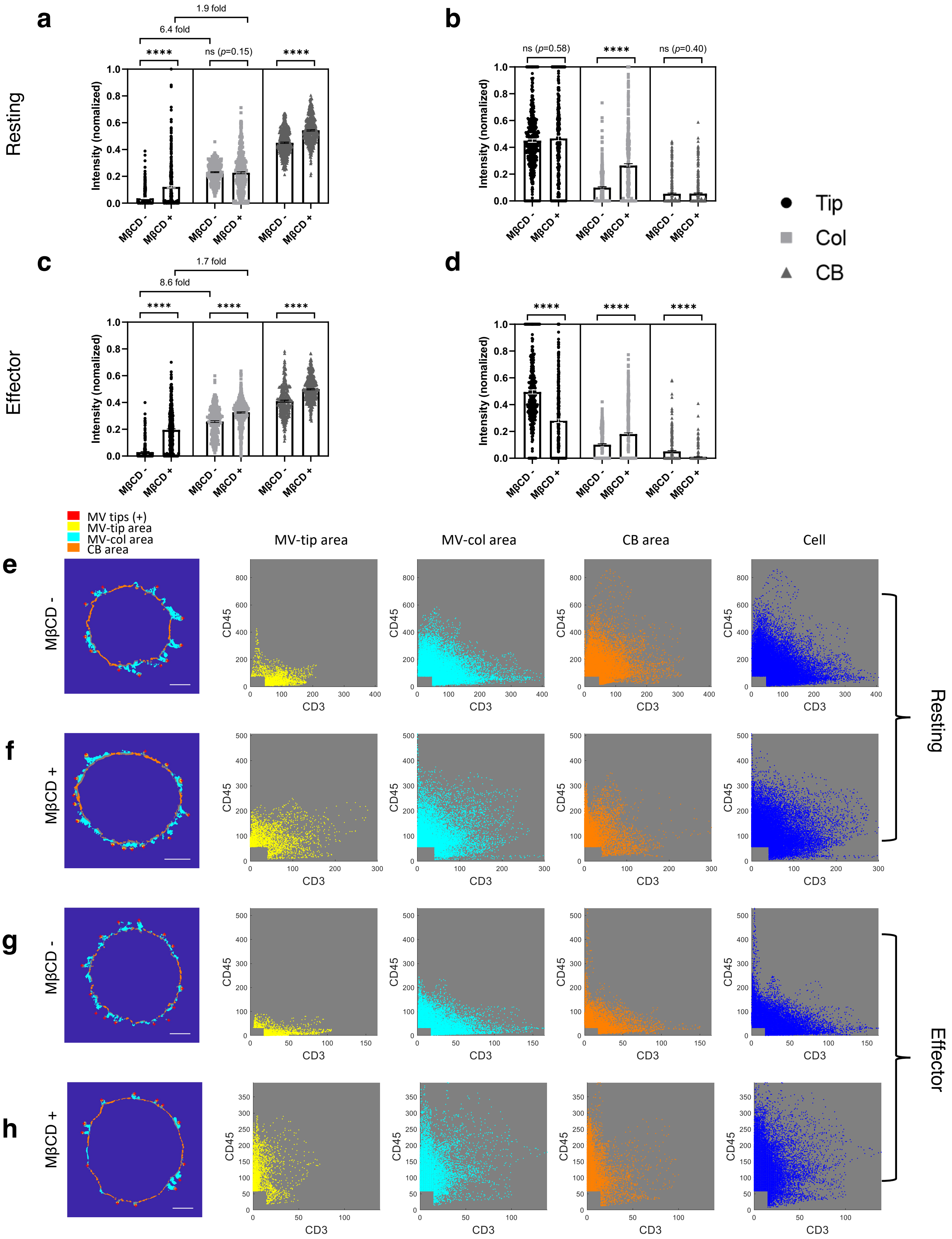

Supplementary Fig. 9 Effect of MβCD on the distribution of CD45 molecules on the surface of human $CD4^+$ T cells. **a-b** The median CD45 (a) and CD3 intensities (b) (normalized) within the segmented areas (MV-tip area (Tip, black circle), MV-col-area (Col, gray square), CB area (CB, dark gray triangle)) of each z-plane images (20 z-plane images per cell) of MβCD untreated (19 cells) or treated (21 cells) resting CD4 T cells. The ratios of the mean CD45 intensities of the MV-col area vs. MV-tip area are indicated (a, top). Each dot represents data collected from a z-plane image. Bars represent the mean and error bars represent the SE. **c-d** The median CD45 (c) and CD3 (d) intensities of MβCD untreated (14 cells) or treated (22 cells) effector T cells similarly analyzed as a-b. *p*-values (^{ns}, $p > 0.05$; ****, $p \leq 0.0001$) were calculated by two tailed Mann Whitney test. **e-h** Segmented maps (left) for the cells shown in Fig.4 a-d, respectively. Images are representative of three independent experiments. The corresponding intensity scatter plots for CD3 and CD45 within the MV-tip area (yellow), MV-col area (cyan), CB area (orange), and entire cell area (Cell, blue) for each segmented map are shown in right 4 panels. Source data for a-d are provided as a Source Data file.

Supplementary Fig. 10

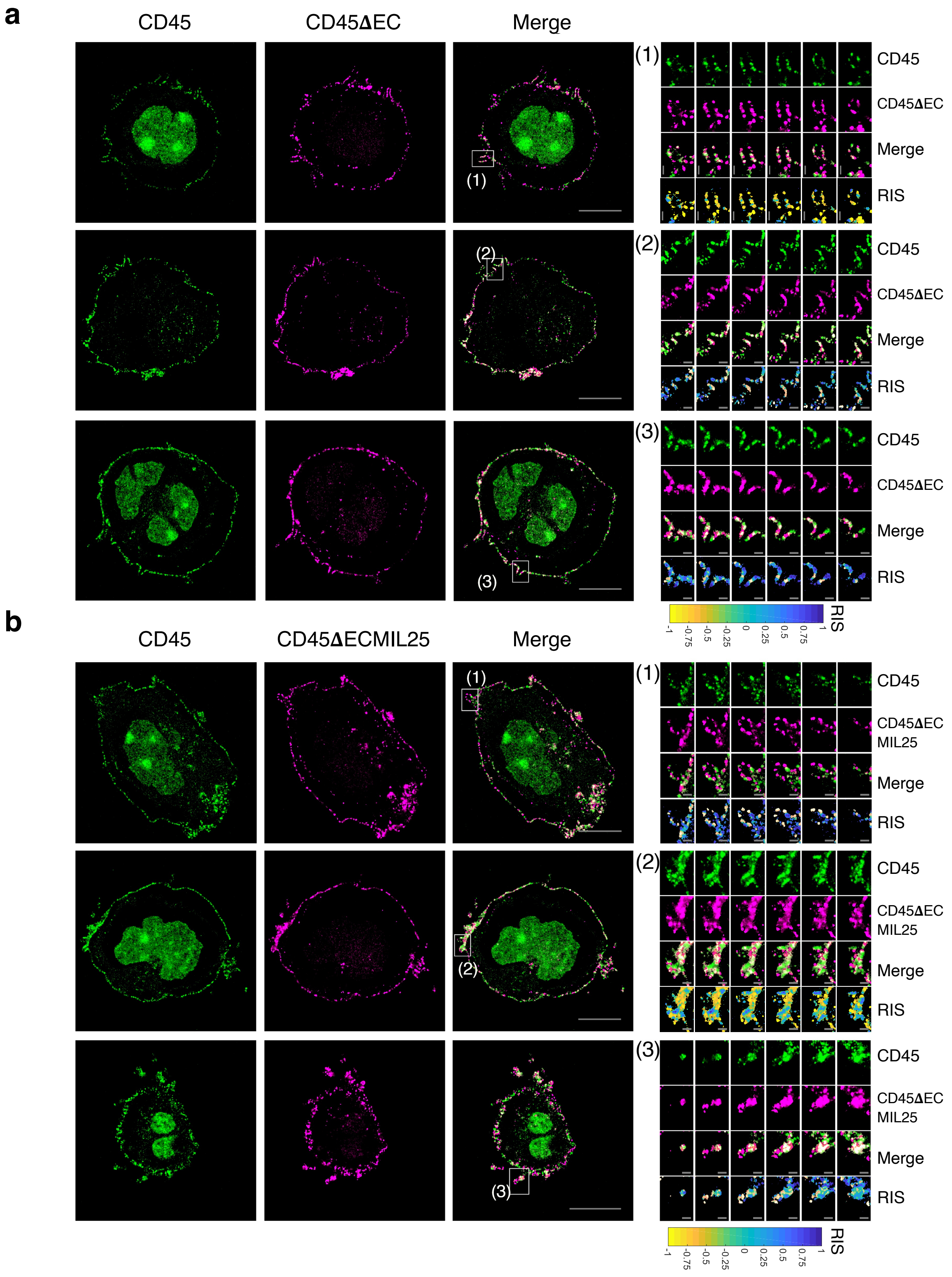

Supplementary Fig. 10. Analysis of Jurkat T cells lentivirally transduced with CD45 mutants. **a** Representative 4x-ExM-Airyscan images of 3 cells out of 15 Jurkat T cells lentivirally transduced with CD45ΔEC mutant. The endogenous CD45 images labeled with anti-CD45-AF488 antibody (green), the membrane expressed mutant images labeled with anti-HA-CF633 antibody (magenta) and the merged images between the two molecules were presented. Scale bars: 5 μm. The six z-stack images (step size 125 nm) of the marked area in the merged images were magnified for each cell (right). Scale bars: 500 nm. The color bar underneath represents RIS values between -1 (100% mutant; 0% endogenous CD45) and +1 (100% endogenous CD45; 0% mutant). **b** Analysis of 3 representative cells out of 25 Jurkat T cells lentivirally transduced with CD45ΔECMIL25 mutant as described as a. Images are representative of three independent experiments.

Supplementary Fig. 11

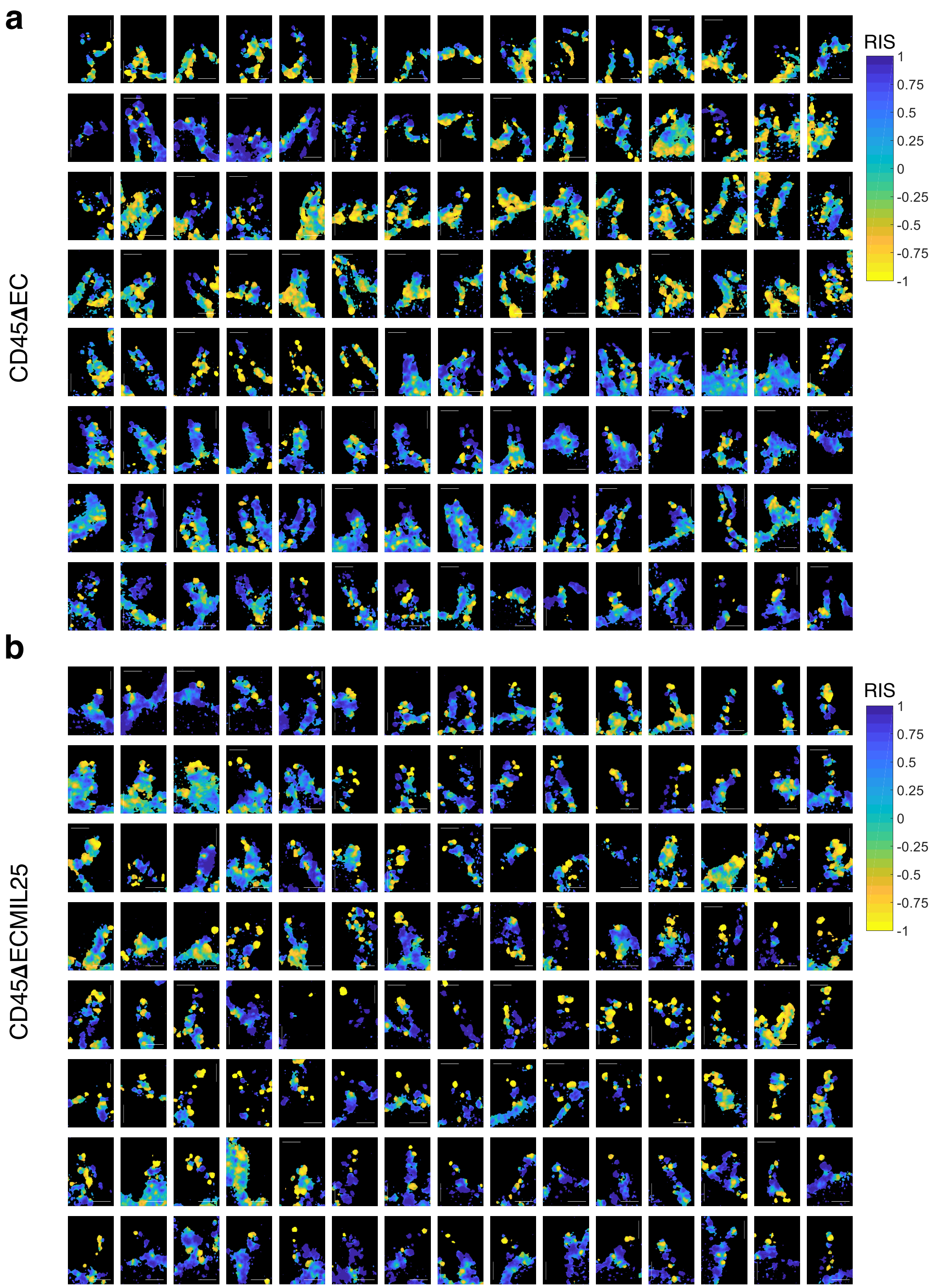

Supplementary Fig. 11. A collection of magnified single MV images of **a** CD45ΔEC (15 cells) or **b** CD45ΔECMIL25 (25 cells) -transduced Jurkat T cells. The color bars on the right represent RIS values between -1 (100% mutant; 0% endogenous CD45) and +1 (100% endogenous CD45; 0% mutant). Scale bars: 500 nm.

Supplementary Fig. 12

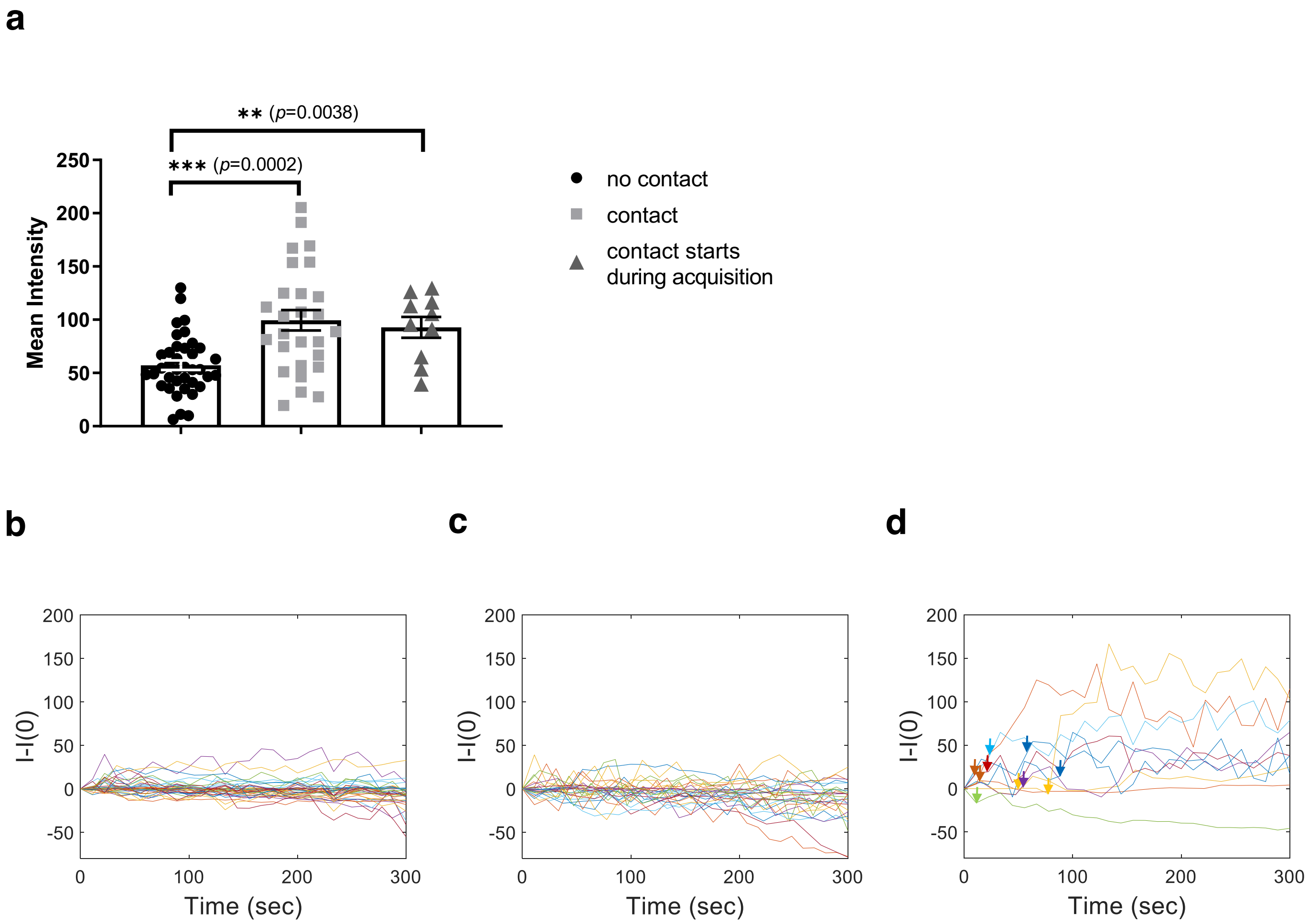

Supplementary Fig. 12. MV-mediated interactions between T cells and APCs induce Ca2+ influx. **a** The mean of the Fluo-4 AM intensities of mouse OTII-*Rag2*$^{-/-}$ CD4^{+} T cells that made no contact with any other cells (no contact, black circle, n=38), persistently made MV-mediated contacts (contact, gray square, n=27) or made contacts later than time zero (contact starts during acquisition, dark gray triangle, n=10) with an antigen pulsed B cell during acquisition time (5min). Each dot represents data collected from a cell. Bars represent the mean and error bars represent the SE. *p*-values (**, $p \leq 0.01$; ***, $p \leq 0.001$) were calculated by two tailed Mann Whitney test. **b** Time trajectories of the Fluo-4 AM Intensity fluctuations (I-I(0)) of OTII-*Rag2*$^{-/-}$ CD4^{+} T cells (n=38) that made no contact with antigen pulsed B cells during acquisition. **c** Time trajectories of the Fluo-4 AM Intensity fluctuations (I-I(0)) of OTII-*Rag2*$^{-/-}$ CD4^{+} T cells persistent-contacting with an antigen pulsed B cell (n=27) during acquisition. **d** Time trajectories of the Fluo-4 AM Intensity fluctuations (I-I(0)) of OTII-*Rag*2$^{-/-}$ CD4^{+} T cell that made contact with an antigen pulsed B cell at any time later than time zero during acquisition (n=10). Arrows indicate initial contact points. Each line in b-d represents data collected from a cell. Source data for a-d are provided as a Source Data file.

Supplementary Note

The segment correlation coefficient (SR′) is calculated between two-channel in a certain segmented area to present correlation within that area as $\mathrm{SR}' = \frac{\sum_{j=1}^{m}(x_j - \bar{X})(y_j - \bar{Y})}{\sqrt{\sum_{j=1}^{m}(x_j - \bar{X})}^2 \sqrt{\sum_{j=1}^{m}(y_j - \bar{Y})}^2}$ (Supplementary Table 1, Equation (3)), where, m is the number of data points within a segmented area, x_j in channel 1 (ch1) and y_j in channel 2 (ch2) are the individual data points indexed with j in a set of segmented data, $\bar{X}$ and $\bar{Y}$ are the mean of total data set of X and of Y, respectively. This quantifies the contribution of negative or positive correlation of a certain segmented area to the whole image. For instance, in Supplementary Fig. 4a, ch1 and ch2 intensities in the region marked blue in Supplementary Fig. 4b were identical, while the values within the area marked yellow were randomly shuffled and reassigned. The Pearson's correlation coefficient, R′, between the ch1 and the ch2 was 0.4. The SR′ values calculated between two channels segmented as in Supplementary Fig. 4f successfully present where the identical regions between the two images are (Supplementary Fig. 4c). The mean result of the 100,000 data sets similarly generated as the Supplementary. Fig 4a showed that the SR′ within the blue marked area was 1 while the SR′ within the yellow marked area became close to zero. (Supplementary Fig. 4d). Supplementary Fig. 4g showed an example image of a human CD4 T cell labeled with anti-CD3 and anti-CD45. The data sets of CD45 and CD3 were segmented by distance from the MV tips (red *) marked in the RIS image in the Supplementary Fig. 4g with a step-size of 12.5 nm. The mean SR′ as a function of distance from the MV tips calculated frame by frame of 20 frames of the cell presented in Supplementary Fig. 4g showed that CD45 and CD3 were negatively correlated near to the MV-tips (Supplementary Fig. 4j, blue plot) while R′ failed to present it (Supplementary Fig. 4h). Next, we segmented the same data set by RIS values with a step-size of 0.05 and calculated the SR′. It showed the SR′ became negative where the RIS values were near -1 or 1; while the SR′ became 1 where the RIS was zero. (Supplementary Fig. 4k, red-vertical line). When the data were randomly shuffled and reassigned, the mean SR′ became close to zero. (Supplementary Fig. 4j, k, orange plots). These results demonstrated that SR′ successfully displays a correlation between two channels within the segmented area and it is sensitive to the RIS between the channels.

Supplementary Tables

Supplementary Table 1. Formulas used to calculate RIS, R′, SR′, MOC and MCC.

abbreviation	Full Name	Equation	
RIS	Ratiometric intensity score [1]	Equation (1) $$RIS = (X_i - Y_i\ (\bar{X}/\bar{Y}))/(X_i + Y_i(\bar{X}/\bar{Y}))$$	X_i *and* Y_i represent the fluorescence intensity of each pixel indexed i for channel X and channel Y, respectively. $\bar{X}$ and $\bar{Y}$ represent the mean of total X and Y, respectively.
R′	Pearson's correlation coefficient [2]	Equation (2) $$R' = \frac{\sum_{i=1}^{n}(X_i - \bar{X})(Y_i - \bar{Y})}{\sqrt{\sum_{i=1}^{n}(X_i - \bar{X})^2}\sqrt{\sum_{i=1}^{n}(Y_i - \bar{Y})^2}}$$	*n* is the total number of data points, X_i and Y_i and are the individual data points indexed with i. $\bar{X}$ and $\bar{Y}$ represent the mean of total X and Y, respectively.
SR′	Segment-correlation coefficient	Equation (3) $$SR' = \frac{\sum_{j=1}^{m}\left(x_j - \bar{X}\right)\left(y_j - \bar{Y}\right)}{\sqrt{\sum_{j=1}^{m}\left(x_j - \bar{X}\right)^2}\sqrt{\sum_{j=1}^{m}\left(y_j - \bar{Y}\right)^2}}$$	*m* is the number of data points within a segmented area. x_j and y_j and are the individual data points indexed with j in a set of segmented data, $\bar{X}$ and $\bar{Y}$ represent the mean of total X and Y, respectively.
MOC	Manders' overlap coefficient [3]	Equation (4) $$MOC = \frac{\sum_{i=1}^{n}(x_i)(y_i)}{\sqrt{\sum_{i=1}^{n}(x_i)^2}\sqrt{\sum_{i=1}^{n}(y_i)^2}}$$	*n* is the total number of pixels, where x_i and y_i refer to the intensities of above-threshold pixel value indexed i in the *X* and *Y* channel images.
MCC_1 and MCC_2	Manders' correlation coefficients 1 and 2 [3]	Equation (5) $$MCC_1 = \frac{\sum_{i=1}^{n}(x_i\ \text{col})}{\sum_{i=1}^{n}(x_i)}$$ $$MCC_2 = \frac{\sum_{i=1}^{n}(y_i\ \text{col})}{\sum_{i=1}^{n}(y_i)}$$	*n* is the total number of pixels in each image being analyzed, x_i col is the pixel intensity of channel *X* indexed i where the corresponding pixel intensity of channel *Y* is above the threshold, y_i col is the pixel intensity of channel *Y* indexed i where the corresponding pixel intensity of channel *X* is above the threshold.
In Supplementary Figs 2,3, and 6, channels *X* and *Y* correspond to CD45 and L-sel respectively; In Figs 2 and 4, and Supplementary Fig. 4, channels *X* and *Y* correspond to CD45 and CD3 respectively; in Fig. 3 and Supplementary Fig.7, channel *X* corresponds to CD45 and channel *Y* corresponds to IgM or IgD respectively; in Fig. 6, and Supplementary Figs. 10 and 11, channel *X* corresponds to the endogenous CD45 and channel *Y* corresponds to CD45ΔEC or CD45ΔECMIL25 mutants.			

Supplementary Table 2. TM domains and the MILs of major cell surface molecules expressed on T cells, B cells or APCs.

Protein	Sequences	TM Domain	Spacer length (a.a.)	MIL
CD45	LHHST SYNSK **ALIAFLAFLIIVTSIALLVVL** YKIYD LHKKR	21	1	22
CD43	PFRNP DENSR **GMLPVAVLVALLAVIVLVALLLL** WRRRQ KRRTG	23	1	24
CD148	LPQDP GVICG **AVFGCIFGALVIVTVGGFIFW** RKKRK DAKNN	21	6	27
L-sel	FSMIK EGDYN **PLFIPVAVMVTAFSGLAFIIWLA** RRLKK GKKSK	23	2	25
CD44	GPIRT PQIPE **WLIILASLLALALILAVCIAV** NSRRR CGQKK	21	2	23
TCRα	DTNLN FQNLS **VIGFRILLLKVAGFNLLMTLRL** WSS	22*	12	34
TCRβ	D CGFTS VSYQQ GVLSA **TILYEILLGKATLYAVLVSALV** LMAMV KRKDF	22*	20	42
CD3δ	CVELD PATVA **GIIVTDVIATLLLALGVFCFA** GHETG RLSGA	21	6	27
CD3ε	RVCEN CMEMD **VMSVATIVIVDICITGGLLLLVYYWS** KNRKA KAKPV	26	0	26
CD3γ	CIELN AATIS **GFLFAEIVSIFVLAVGVYFIA** GQDGV RQSRA	21	9	30
CD3ζ	AQSFG LLDPK **LCYLLDGILFIYGVILTALFLRV** KFSRS ADA	23*	0	23
CD4	KV LPTWS TPVQP **MALIVLGGVAGLLLFIGLGIFF** CVRCR HRRRQ	22	13	35
CD8a	HTRGL DFACD **IYIWAPLAGTCGVLLLSLVIT** LYCNH RNRRR	21	4	25
CD8b	ETQKG PLCSP **ITLGLLVAGVLVLLVSLGVAI** HLCCR RRRAR	21	6	27
CD2	PVSCP EKGLD **IYLIIGICGGGSLLMVFVALLVFYIT** KRKKQ RSRRN	26	0	26
CD28	SPLFP GPSKP **FWVLVVVGGVLACYSLLVTVAFIIFWV** RSKRS RLLHS	27	1	28
CTLA4	IDPEP CPDSD **FLLWILAAVSSGLFFYSFLLT** AVSLS KMLKK	21	5	26
PD1	RPAGQ FQTLV **VGVVGGLLGSLVLLVWVLAVI** CSRAA RGTI	21	11	32
CD25	ETSIF TTEYQ **VAVAGCVFLLISVLLLSGL** TWQRR QRKSR	19	5	24
CD122	TKPAA LGKDT **IPWLGHLLVGLSGAFGFIILVYLLI** NCRNT GPWLK	25	3	28
LAT	MEEA **ILVPCVLGLLLLPILAMLMALCV** HCHRL PGSYD	23	1	24
CD11a	VDVVY EKQML **YLYVLSGIGGLLLLLLIFIVL** YKVGF FKRNL	21	4	25
CD18	ESREC VAGPN **IAAIVGGTVAGIVLIGILLLVIW** KALIH LSDLR	23	6	29
PSGL1	APDHI SVKQC **LLAILILALVATIFFVCTVVL** AVRLS RKGHM	21	4	25
IL6R	VQDSS SVPLP **TFLVAGGSLAFGTLLCIAIVL** RFKK T WKLRA	21	7	28
CD40L	AATGL PISMK **IFMYLLTVFLITQMIGSALFAVYL** HRRLD KIEDE	24	0	24
CD86	DPQPP PDHIP **WITAVLPTVIICVMVFCLILW** KWKKK KRPRN	21	2	23
CD80	TTKQE HFPDN **LLPSWAITLISVNGIFVICCL** TYCFA PRCRE	21	7	28
LFA3	IPSSG HSRHR **YALIPIPLAVITTCIVLYMNGIL** KCDRK PDRTN	23	0	23
CD79a	LDMGE GTKNR **IITAEGIILLFCAVVPGTLLLF** RKRWQ NEKLG	22	0	22
CD79b	QLKQR NTLKD **GIIMIQTLLIILFIIVPIFLL** LDKDD SKAGM	21	1	22
CD19	WLLR TGGWK **VSAVTLAYLIFCLCSLVGILHL** QRALV LRRKR KR	22	1	23
CD40	VVCGP QDRLR **ALVVIPIIFGILFAILLVLVFI** KKVAK KPTNK	22	0	22
ICAM1	TVNVL SPRYE **IVIITVVAAAVIMGTAGLSTYLY** NRQRK IKKYR	23	1	24
CD74	PGAPE SKCSR **GALYTGFSILVTLLLAGQATTAYFLY** QQQGR LDKLT	26	4	30
HLA-DRA	APSPL PETTE **NVVCALGLTVGLVGIIIGTIFII** KGVRK SNAAE	23	0	23
HLA-DRB1	RARSE SAQSK **MLSGVGGFVLGLLFLGAGLFIYF** RNQKG HSGLQ	23	0	23
CD22	YYSPE TIGRR **VAVGLGSCLAILILAICGL** KLQRR WKRTQ	19	0	19
CD33	KQETR AGVVH **GAIGGAGVTALLALCLCLIFFIV** KTHRR KAART	23	0	23
CD64	LQLPT PVWFH **VLFYLAVGIMFLVNTVLWVTI** RKELK RKKKW	21	0	21
CD32a	KPVT ITVQV PSMGS SSPMG **IIVAVVIATAVAAIVAAVVALIY** CRKKR ISANS	23	19	42
CD32b	KPVTI TVQAP **SSSPMGIIVAVVTGIAVAAIVAA** VVALI YCRKK	23	16	39
CD32c	K PVTIT VQAPS SSPMG **IIVAVVTGIAVAAIVAAVVALIY** CRKKR ISANS	23	16	39
CD16a	ETVNI TITQG LAVST ISSFF PPGYQ **VSFCLVMVLLFAVDTGLYFSV** KTNIR	21	24	45
CR2	NPPLA VCRSR **SLAPVLCGIAAGLILLTFLIVITLYVIS** KHRAR NYYTD	28	0	28
CR1	LAKCT SRTHD **ALIVGTLSGTIFFILLIIFLSWIIL** KHRKG NNAHE	25	0	25
**Mean**		**22.5**	**4.9**	**27.4**
**SD**		**2.0**	**6.1**	**5.9**

Amino acid sequences of the TM domain (underline) plus flanking residues are shown. The nearest charged a.a. residues [lysine (K), arginine (R), glutamic acid (E), aspartic acid (D), histidine (H)] located at each end of the TM domain are shown in red. The length of the TM domain, spacer, and MIL for each protein are shown on the right. The length of TM domains predicted in the Universal Protein Resource (UniProt; https://www.uniprot.org/) or reported in references [4,5] are asterisked. CD45 was excluded from the mean and SD calculation.

Supplementary Table 3. Homologies of the TM domains of 38 CD45 proteins.

Species	Sequences
Homo Sapiens (Human)	LHHST SYNS**K** **ALIAFLAFLIIVTSIALLVVLY** **K**IYDL HKKRS
Anas platyrhynchos (Mallard)	GSIKT KYNS**R** **ALIIFLVFLIVVTSIALLLVLY** **K**IYDL HQKKL
Anolis carolinensis (Green anole)	ETVNT KYNA**K** **ALIGFLAFLIIVTSLALLIVLY** **R**IYTL NRQKS
Aotus nancymaae (Ma's night monkey)	QFQST SYNS**K** **ALIAFLAFLIIVTSIALLVVLY** **K**IYDL HKKRS
Aotus nigriceps (Black-headed night monkey)	QFQST SYNF**K** **ALIAFLAFLIIVTSIALLVVLY** **K**IYDL HKKRS
Aotus vociferans (Spix's owl monkey)	LHHST SYNS**K** **ALIAFLAFLIIVTSIALLVVLY** **K**IYDL HKKRS
Bos taurus (Bovine)	VTAST SYNA**K** **ALIIFLVFLIIVTSIALLIVLY** **K**IYDL HKKRS
Callithrix jacchus (White-tufted-ear marmoset)	KFQST TYNS**K** **ALIAFLAFLIIVTSIALLVVLY** **K**IYDL HKKRS
Canis lupus familiaris (Dog)	FSHTT SYNS**R** **ALIIFLVFLIIVTFLALLVVLY** **K**IYDL RNKRS
Cavia porcellus (Guinea pig)	EFYTT SYNS**K** **ALIAFLVFLIIVTSVALLVVLY** **K**IYDL HKKRS
Cebus capucinus imitator (Panamanian white-faced capuchin)	KAKST SFNS**K** **ALIAFLAFLIVVTSIALLLVLY** **K**IYDL HKKRS
Cercocebus atys (Sooty mangabey)	LHEST SYNS**K** **ALIAFLAFLIIVTSIALLVVLY** **K**IYDL HKKRS
Chlorocebus sabaeus (Green monkey)	LHEST SYNS**K** **ALIAFLAFLIIVTSIALLVVLY** **K**IYDL HKKRS
Colobus angolensis palliatus (Peters' Angolan colobus)	VDEPT SYNS**K** **ALIAFLAFLIIVTSIALLVVLY** **K**IYDL HKKRS
Cyprinus carpio (Common carp)	ATHST RYND**K** **ALIGFLVFLILVTSLALLFVLY** **K**LFLL KRKRT
Danio rerio (Zebrafish)	ASIST SYNE**K** **AVIGFLAFLIIVTSVALLFVLY** **K**IFLL KRKRT
Felis catus (Cat)	VFHST SYNS**K** **ALIAFLVFLIIVTFLALLFVLY** **K**IYDL RNRRS
Ficedula albicollis (Collared flycatcher)	ESIKT RYNS**R** **ALIIFLAFLIIVTVIALLLVLY** **K**IYDL HKKKL
Gallus gallus (Chicken)	EEIYT RYNS**R** **ALIIFLVFLIVVTSIALLLVLY** **K**IYDL HQKKL
Gorilla gorilla (Western Gorilla)	LHQST SYNS**K** **ALIAFLAFLIIVTSIALLVVLY** **K**IYDL HKKR
Ictidomys tridecemlineatus (Squirrel)	KHHST SYNS**K** **ALIAFLAFLIVVTSIALLVVLY** **K**IYDL HKKRS
Loxodonta africana (African elephant)	RTKST SYNS**K** **ALIIFLVFLIIVTSIALLFVLY** **K**IYDL HKKRS
Macaca mulatta (Rhesus macaque)	LREST SYNS**K** **ALIAFLAFLIIVTSIALL-VPI** **H**ADIL LETYK
Macaca nemestrina (Pig-tailed macaque)	LREST SYNS**K** **ALIAFLAFLIIVTSIALLVVLY** **K**IYDL HKKRS
Meleagris gallopavo (Wild turkey)	KEIYT RYNS**R** **ALIIFLVFLIVVTSIALLLVLY** **K**IYDL RQKKL
Monodelphis domestica (Gray short-tailed opossum)	KSEVT RYNS**H** **ALIMFLIFLIVVTSLALLIVLY** **K**IYDL HKKRS
Mus musculus (Mouse)	RNEST NFNA**K** **ALIIFLVFLIIVTSIALLVVLY** **K**IYDL RKKRS
Mustela putorius furo (European domestic ferret)	VSRST SYNS**R** **ALITFLVFLIIVTFLALLVVLY** **K**IYDL RSKRS
Nomascus leucogenys (Northern white-cheeked gibbon)	LHKST SYNS**K** **ALIAFLAFLIIVTSIALLVVLY** **K**IYDL HKKRS
Oryctolagus cuniculus (Rabbit)	KHQST SYNS**K** **ALIAFLAFLIIVTSIALLVVLY** **K**IYDL HKKRS
Otolemur garnettii (Small-eared galago)	RNQST SYNS**K** **ALIGFLAFLIVVTTIALLAVLY** **K**IYDL HKKRS
Pan paniscus (Pygmy chimpanzee)	LHQST SYNS**K** **ALIAFLAFLIIVTSIALL-VPI** **H**ADIL LETYK
Pan troglodytes (Chimpanzee)	LHQST SYNS**K** **ALIAFLAFLIVVTSIALLVVLY** **K**IYDL HKKRS
Papio anubis (Olive baboon)	LHEST SYNS**K** **ALIAFLAFLIIVTSIALLVVLY** **K**IYDL HKKRS
Pongo abelii (Sumatran orangutan)	LHKST SYNS**K** **ALIAFLTFLIIVTSIALLVVLY** **K**IYDL HKKRS
Rattus norvegicus (Rat)	KPQST SYNS**K** **ALIIFLVFLIIVTSIALLVVLY** **K**IYDL RKKRS
Rhinopithecus bieti (Black snub-nosed monkey)	LPKST SYNS**E** **ALITFLAFLIIVTSIALLVVLY** **K**IYDL HKKRS
Sus scrofa (Pig)	FLVKD LNNS**H** **ALIIFLIFLIIVTSIALLVS**-- **R**VFNM FKDDE

The amino acid sequences of the TM domains (underline) ± flanking residues are shown and the predicted TM domains. Charged TM domain-flanking residues are shown in red. The length of TM domains predicted in the Universal Protein Resource (UniProt; https://www.uniprot.org/)

Supplementary References

1 Yu, W., So, P. T., French, T. & Gratton, E. Fluorescence generalized polarization of cell membranes: a two-photon scanning microscopy approach. *Biophys J* **70**, 626-636, doi:10.1016/S0006-3495(96)79646-7 (1996)

2 Rodgers, J. L. & Nicewander, W. A. Thirteen Ways to Look at the Correlation Coefficient. The American Statistician 42, 59-66, doi:10.2307/2685263 (1988).

3 Manders, E. M. M., Verbeek, F. J. & Aten, J. A. Measurement of co-localization of objects in dual-colour confocal images. Journal of Microscopy 169, 375-382, doi:10.1111/j.1365-2818.1993.tb03313.x (1993).

4 Call, M. E. et al. The Structure of the ζζ Transmembrane Dimer Reveals Features Essential for Its Assembly with the T Cell Receptor. Cell 127, 355-368, doi:https://doi.org/10.1016/j.cell.2006.08.044 (2006).

5 Krshnan, L., Park, S., Im, W., Call, M. J. & Call, M. E. A conserved αβ transmembrane interface forms the core of a compact T-cell receptor–CD3 structure within the membrane. Proceedings of the National Academy of Sciences 113, E6649, doi:10.1073/pnas.1611445113 (2016).
