# Supplementary material for: CD45 pre-exclusion from the tips of T cell microvilli prior to antigen recognition
Source: Nat Commun. 2021 Jun 23;12:3872. doi: 10.1038/s41467-021-23792-8 (PMC8222282; doi:10.1038/s41467-021-23792-8)
Supplement: Supplementary file 3 — Descriptions of Additional Supplementary Files [file 41467_2021_23792_MOESM3_ESM.pdf]

Descriptions of Additional Supplementary Files

Supplementary Movie 1
Description: A representative series of z-stack 4x-ExMAiryscan images of a human CD4+ T cell selected among 35 cells labelled with anti-CD45-AF488 (green, left), anti-CD3-CF633 (magenta; middle) and the merged image (right) with a step size of 62.5nm per frame. Scale bar in the merged image: 2 µm.

Supplementary Movie 2
Description: MV-mediated interactions between a mouse OTII-Rag2-/- CD4+ T cells and an antigen pulsed mouse B cell induce Ca2+ influx. A time-lapse of ODT images (A) overlaid with the corresponding Fluo-4 AM images (B). The mean intensities of ROI area (C; magenta) in each frame are plotted in the graph D.

Supplementary Movie 3
Description: MV-mediated interactions between mouse OTIIRag2-/- CD4+ T cells and antigen pulsed mouse B cells induce Ca2+ influx. A time-lapse of ODT images (A) overlaid with the corresponding Fluo-4 AM images (B). The mean intensities of each ROI areas marked in C are plotted in the graph D.

Supplementary Movie 4
Description: MV-mediated interactions between mouse OTIIRag2-/- CD4+ T cells and mouse B cells (no antigen pulsed) induce Ca2+ influx. A time-lapse of ODT images (A) overlaid with the corresponding Fluo-4 AM images (B). The mean intensities of each ROI areas marked in C are plotted in the graph D.
