# Supplementary material for: CD45 pre-exclusion from the tips of T cell microvilli prior to antigen recognition
Source: Nat Commun. 2021 Jun 23;12:3872. doi: 10.1038/s41467-021-23792-8 (PMC8222282; doi:10.1038/s41467-021-23792-8)
Supplement: Supplementary file 8 — Reporting Summary [file 41467_2021_23792_MOESM8_ESM.pdf]

nature research

Corresponding author(s): Yunmin Jung
Last updated by author(s): Apr 23, 2021

Reporting Summary

Nature Research wishes to improve the reproducibility of the work that we publish. This form provides structure for consistency and transparency in reporting. For further information on Nature Research policies, see our Editorial Policies and the Editorial Policy Checklist.

Statistics

For all statistical analyses, confirm that the following items are present in the figure legend, table legend, main text, or Methods section.

n/a	Confirmed	
☐	☒	The exact sample size (n) for each experimental group/condition, given as a discrete number and unit of measurement
☒	☐	A statement on whether measurements were taken from distinct samples or whether the same sample was measured repeatedly
☐	☒	The statistical test(s) used AND whether they are one- or two-sided<br>*Only common tests should be described solely by name; describe more complex techniques in the Methods section.*
☒	☐	A description of all covariates tested
☒	☐	A description of any assumptions or corrections, such as tests of normality and adjustment for multiple comparisons
☐	☒	A full description of the statistical parameters including central tendency (e.g. means) or other basic estimates (e.g. regression coefficient) AND variation (e.g. standard deviation) or associated estimates of uncertainty (e.g. confidence intervals)
☐	☒	For null hypothesis testing, the test statistic (e.g. F, t, r) with confidence intervals, effect sizes, degrees of freedom and P value noted<br>*Give P values as exact values whenever suitable.*
☒	☐	For Bayesian analysis, information on the choice of priors and Markov chain Monte Carlo settings
☒	☐	For hierarchical and complex designs, identification of the appropriate level for tests and full reporting of outcomes
☐	☒	Estimates of effect sizes (e.g. Cohen's d, Pearson's r), indicating how they were calculated

Our web collection on statistics for biologists contains articles on many of the points above.

Software and code

Policy information about availability of computer code

Data collection	ZEN Black 2.3 SP1 FP2 (Carl Zeiss, Version 14.0.16.201); Nanoimager software (ONI, Version 1.1.6165-012f4ed3); Tomostudio software (Tomocube, Version HT-2H-2.7.35); FACSDiva (BD Biosciences, version 8.0.1).
Data analysis	Matlab (MathWorks, R2018a); Prism 8 & 9 (GraphPad, Version 8.4.1 & Version 9.0.1); ZEN Black 2.3 SP1 FP2 (Carl Zeiss, Version 14.0.16.201); Nanoimager software (ONI, Version 1.1.6165-012f4ed3); Image J (version 1.52p); Tomostudio software (Tomocube, Version HT-2H-2.7.35); FlowJo (BD, v10.4.2.& v10.6.2). TMHMM v2.0 (transmembrane helices based on a hidden Markov model (http://www.cbs.dtu.dk/services/TMHMM/); Custom codes used in this study are available (https://github.com/ymjung1/ncomms_2021_Jung).

For manuscripts utilizing custom algorithms or software that are central to the research but not yet described in published literature, software must be made available to editors and reviewers. We strongly encourage code deposition in a community repository (e.g. GitHub). See the Nature Research guidelines for submitting code & software for further information.

Data

Policy information about availability of data

All manuscripts must include a data availability statement. This statement should provide the following information, where applicable:

- Accession codes, unique identifiers, or web links for publicly available datasets
- A list of figures that have associated raw data
- A description of any restrictions on data availability

Source data are provided with this paper. Data that are generated and used in this study are available from yjung@lji.org upon reasonable request. The sequences and the predicted lengths of transmembrane domains listed in Supplementary Table 2 and 3 are available in in the Universal Protein Resource (UniProt; https://www.uniprot.org/) or reported in references 63,64 . The probability of TM domain of CD45 in Fig. 5b was analyzed using TMHMM v2.0 (transmembrane helices based on a hidden Markov model (http://www.cbs.dtu.dk/services/TMHMM/).

Field-specific reporting

Please select the one below that is the best fit for your research. If you are not sure, read the appropriate sections before making your selection.

☒ Life sciences ☐ Behavioural & social sciences ☐ Ecological, evolutionary & environmental sciences

For a reference copy of the document with all sections, see nature.com/documents/nr-reporting-summary-flat.pdf

Life sciences study design

All studies must disclose on these points even when the disclosure is negative.

Sample size	Sample size was chose with considerations on the total time needed for taking the full dataset and obtain enough data for statistic analysis. Please see individual figures for the exact sample size in each case.
Data exclusions	Some cell images that were failed to the segmentation were excluded, for example, due to multi-cells in the same filed of view; Airyscan images that have noticeable sample drifting during the image acquisition were excluded from the data analysis.
Replication	All data was collected from at least 3 independent experiments, except Supplementary Fig 6 (2 independent experiments) and Supplementary Video 4. (1 independent experiment). All attempts at replication were successful.
Randomization	The cells and image field of view were selected randomly for each group of experiments
Blinding	Blinding was performed during image segmentation and analysis. Image analysis was performed automatically.

Reporting for specific materials, systems and methods

We require information from authors about some types of materials, experimental systems and methods used in many studies. Here, indicate whether each material, system or method listed is relevant to your study. If you are not sure if a list item applies to your research, read the appropriate section before selecting a response.

Materials & experimental systems

n/a	Involved in the study	
☐	☒	Antibodies
☐	☒	Eukaryotic cell lines
☒	☐	Palaeontology and archaeology
☐	☒	Animals and other organisms
☐	☒	Human research participants
☒	☐	Clinical data
☒	☐	Dual use research of concern

Methods

n/a	Involved in the study	
☒	☐	ChIP-seq
☐	☒	Flow cytometry
☒	☐	MRI-based neuroimaging

Antibodies

Antibodies used

1) Alexa Fluor® 488 anti-human CD45 Antibody, Biolegend (clone HI30), Cat# 304017, Isotype: Mouse IgG1, κ, RRID:AB_389314.
2) Alexa Fluor® 647 anti-human CD45 Antibody, Biolegend (clone HI30), Cat# 304056, Isotype: Mouse IgG1, κ, RRID:AB_2564155.
3) Alexa Fluor® 488 anti-mouse CD45 Antibody Biolegend (clone 30-F11) ,Cat# 103122, Isotype: Rat IgG2b, κ, RRID:AB_493531.
4) LEAF™ Purified anti-human CD62L Antibody Biolegend (clone DREG-56), Cat# 304812, Isotype: Mouse IgG1, κ, RRID:AB_314472.
5) LEAF™ Purified anti-mouse CD62L Antibody Biolegend (clone MEL-14), Cat# 104416, Isotype: Rat IgG2a, κ, RRID:AB_313101.
6) LEAF™ Purified anti-human CD3 Antibody Biolegend (clone UCHT1), Cat# 300414, Isotype: Mouse IgG1, κ,RRID:AB_314068.
7) Purified anti-human IgM Antibody Biolegend (clone MHM-88), Cat# 314502, Isotype: Mouse IgG1, κ, RRID:AB_493003.
8) Purified anti-human IgD Antibody, Biolegend (clone IA6-2), Cat# 348202,Isotype: Mouse IgG2a, κ, RRID:AB_10550095.
9) Ultra-LEAF™ Purified anti-HA.11 Epitope Tag Antibody Biolegend (clone 16B12), Cat# 901521, Isotype: Mouse IgG1, κ, RRID:AB_2716058.
10) Ultra-LEAF™ Purified anti-human CD3 Antibody Biolegend (clone OKT3), Cat# 317326, Isotype:Mouse IgG2a, κ RRID:AB_11150592.
11) Ultra-LEAF™ Purified anti-human CD28 Antibody Biolegend (clone CD28.2), Cat# 302934, Isotype: Mouse IgG1, κ, RRID:AB_11148949.
12) AF568 -anti-human CD62L (L-selectin; DREG-56, Biolegend) antibody (Custom labeled)
13) CF633-anti-human CD3 (UCHT1, Biolegend) antibody (Custom labeled)
14) CF633-anti-HA.11 epitope tag (16B12, Biolegend) antibody (Custom labeled)
15) CF633-anti-human IgM (MHM-88, Biolegend) antibody (Custom labeled)
16) CF633-anti-human IgD (IA6-2, Biolegend) antibody(Custom labeled)
17) CF633-anti-mouse CD62L (MEL-14, Biolegend) antibody (Custom labeled)

Validation	Each of these antibody were tested by immunofluorescent staining with flow cytometric analysis with mouse splenocytes or human peripheral blood lymphocytes (from manufacturer). Images or flow cytometric analysis of cells labeled with AF488-conjugated isotype-control antibodies (Alexa Fluor® 488 Mouse IgG1, κ Isotype Ctrl (clone MOPC-21, cat# 400129); Alexa Fluor® 488 Rat IgG2b, κ Isotype Ctrl Antibody (clone RTK4530, cat# 400625;) or CF633 conjugated (using Mix-n-Stain™ CF™ 633 Antibody Labeling Kit (sigma, MX633S100-1KT)) isotype control antibodies (LEAF™ Purified Mouse IgG1, κ Isotype Ctrl Antibody; (clone MOPC-21, cat# 400124); LEAF™ Purified Rat IgG2b, κ Isotype Ctrl Antibody, (clone RTK4530, cat# 400622)) were tested for comparison. For 9, the antibody was tested by western blot with total cell lysate (15 μg protein) from CHO and CHO stably transfected with HA tag fused protein. (from manufacturer)

Eukaryotic cell lines

Policy information about cell lines

Cell line source(s)	Jurkat T cells (Clone E6-1 (ATCC), gift from Dr. Zachary Katz , La Jolla Institute for Immunology); 293T cells (ATCC)
Authentication	The cell line was not authenticated.
Mycoplasma contamination	The cell line was not tested for mycoplasma contamination.
Commonly misidentified lines (See ICLAC register)	The name of the cell line is not commonly misidentified.

Animals and other organisms

Policy information about studies involving animals; ARRIVE guidelines recommended for reporting animal research

Laboratory animals	C57BL/6J mice (male or female, 11-33 weeks old). Foxp3YFP-Cre reporter mice (male, 12-13 weeks old). OT-II x Rag2-/- mice (male or female, 11~31 weeks old)
Wild animals	This study did not involve wild animals.
Field-collected samples	This study did not involve field-collected samples
Ethics oversight	Mice were maintained and used by following the guidelines of the La Jolla Institute for Immunology Animal Care and Use Committee, and approval for use of mice was obtained from the La Jolla Institute for Immunology according to criteria outlined in the Guide for the Care and Use of Laboratory Animals from the National Institutes of Health.

Note that full information on the approval of the study protocol must also be provided in the manuscript.

Human research participants

Policy information about studies involving human research participants

Population characteristics	Age, gender,and race are unspecified.
Recruitment	Human PBMCs were obtained from healthy donors through the La Jolla Institute for Immunolog Clinical Core in accordance with the La Jolla Institute for Immunology Normal Blood Donor Program (VD-057) approved by the La Jolla Inst for Allergy & Immunology IRB #1 (IRB registration number: IRB0000850; federalwide assurance number: FWA00000032).
Ethics oversight	For blood obtained through the LJI Normal Blood Donor Program, the program undergoes ethical review by La Jolla Institute for Immunology Review Board (La Jolla Institute for Immunology IRB), in accordance with Dept of Health and Human Services Policy for Protection of Human Research Subjects (45 CFR 46). The current IRB protocol number is VD-057-0216 and the Institute's federal wide assurance number for human subjects protection is FWA 00000032.

Note that full information on the approval of the study protocol must also be provided in the manuscript.

Flow Cytometry

Plots

Confirm that:

- [x] The axis labels state the marker and fluorochrome used (e.g. CD4-FITC).
- [x] The axis scales are clearly visible. Include numbers along axes only for bottom left plot of group (a 'group' is an analysis of identical markers).
- [ ] All plots are contour plots with outliers or pseudocolor plots.
- [x] A numerical value for number of cells or percentage (with statistics) is provided.

Methodology

Sample preparation	For the viability test of the MβCD-treated cells, human CD4+ were purified from PBMCs prepared from the whole blood of healthy donors using EasySep negative selection kits (StemCell Technologies). The MβCD-treated cells were stained with 7-

AAD (20 μg/ml, BD Biosciences) for 20 min on ice, followed by washing with cold 1% FBS/PBS. The cells were resuspended in 200 μl of 1% FBS/PBS and kept on ice in the dark. Cells were immediately loaded for flow cytometry acquisition.

For sorting the mouse Treg cells, mouse CD4+ cells isolated from Foxp3YFP-Cre mice using EasySep negative selection kits (StemCell Technologies) were labeled with Alexa Flour 488-conjugated anti-mouse CD45 and CF633-conjugated anti-mouse L-selectin on ice for 20 min as described in Method. Cells were washed and fixed and were resuspended in 0.5 ml PBS and were kept at 4°C until sorting.

For sorting the Jurkat T cells transduced with CD45 mutants CD45ΔEC, CD45ΔECMIL25, cells were labeled with Alexa Flour 488-conjugated anti-human CD45 and CF633-conjugated anti-HA as described in Method. Cells were washed and fixed and were resuspended in 0.5 ml PBS and were kept at 4°C until sorting.

Instrument

Flow cytometry data was collected on a BD FACSCelesta Flow Cytometer (BD Bioscience), and sorting was performed on a BD FACSAria II Cell Sorter or a BD FACSAria Fusion Cell Sorter (BD Bioscience).

Software

Data aquisition and sorting was performed using FACSDiva software (BD Biosciences), and flow cytometry data analysis was done with FlowJo (BD Biosciences) software.

Cell population abundance

CD4+ Treg cell population in the Foxp3-YFP+, CD45+, Lsel+ sorted is up to 96% purity provided by the manufacture of the EasySep mouse CD4+ negative selection kit. Jurkat T cells expressing HA+ cells were expected to be 100% since the top 10% of high level of HA were gated.

Gating strategy

For the The viability test of the MβCD-treated cells, single cells gated using forward versus side scatter (FSC vs. SSC) were analyzed for 7-AAD fluorescence. Unstained control sample was used for gating.

For sorting the Treg cells, after selecting single cells determined by FSC vs. SSC gating, CD45+Lsel+YFP+ cells were sorted and collected. Fluorescence minus one (FMO) controls for CD45 and Lsel were used for the gating.

For sorting the Jurkat T cells transduced with CD45 mutants, single cells determined by FSC vs. SSC gating. CD45+ and the top 10% of the high level HA expressed cells were gated. Untransduced control Jurkat cells labeled with Alexa Flour 488-conjugated anti-human CD45 and CF633-conjugated anti-HA were used for gating.

☒ Tick this box to confirm that a figure exemplifying the gating strategy is provided in the Supplementary Information.
